# Computation of adherence to medication and visualization of medication histories in R with *AdhereR*: Towards transparent and reproducible use of electronic healthcare data

**DOI:** 10.1371/journal.pone.0174426

**Published:** 2017-04-26

**Authors:** Alexandra Lelia Dima, Dan Dediu

**Affiliations:** 1 Amsterdam School of Communication Research ASCoR, University of Amsterdam, Amsterdam, the Netherlands; 2 Health Services and Performance Research (HESPER EA 7425), University Claude Bernard Lyon 1, Lyon, France; 3 Language and Genetics Department, Max Planck Institute for Psycholinguistics, Nijmegen, The Netherlands; 4 Donders Institute for Brain, Cognition and Behavior, Radboud University, Nijmegen, The Netherlands; Universite de Bretagne Occidentale, FRANCE

## Abstract

Adherence to medications is an important indicator of the quality of medication management and impacts on health outcomes and cost-effectiveness of healthcare delivery. Electronic healthcare data (EHD) are increasingly used to estimate adherence in research and clinical practice, yet standardization and transparency of data processing are still a concern. Comprehensive and flexible open-source algorithms can facilitate the development of high-quality, consistent, and reproducible evidence in this field. Some EHD-based clinical decision support systems (CDSS) include visualization of medication histories, but this is rarely integrated in adherence analyses and not easily accessible for data exploration or implementation in new clinical settings. We introduce *AdhereR*, a package for the widely used open-source statistical environment R, designed to support researchers in computing EHD-based adherence estimates and in visualizing individual medication histories and adherence patterns. *AdhereR* implements a set of functions that are consistent with current adherence guidelines, definitions and operationalizations. We illustrate the use of *AdhereR* with an example dataset of 2-year records of 100 patients and describe the various analysis choices possible and how they can be adapted to different health conditions and types of medications. The package is freely available for use and its implementation facilitates the integration of medication history visualizations in open-source CDSS platforms.

## Introduction

Electronic healthcare data (EHD) are a main source of information on patients’ adherence to medications for research and clinical practice [1]. As adherence impacts considerably on health outcomes and cost-effectiveness of healthcare [2], EHD have the potential to support large-scale investigations of the prevalence and role of adherence in routine care, as well as provide the infrastructure for implementing adherence-enhancing programmes. Routinely-collected information on medication dispensing and/or prescribing is nowadays accessible in primary or secondary care electronic medical records (EMR), pharmacy dispensing databases, or health insurance claims systems. These data can be used to estimate retrospectively how patients used the recommended medication over specific intervals (to the extent that obtaining a medication supply led to medication use) without the intrusiveness and costs inherent to prospective data collection via self-report or electronic monitoring devices. EHD are an objective and low-cost solution to measuring adherence in large patient samples [1,3]. Yet, concerns have been raised regarding the lack of standardization and transparency in data analysis, which make it difficult to ascertain research quality and to compare studies when synthesizing available evidence [4,5].

Several notable efforts to systematize definitions and operationalizations of adherence to medications have already had a benefic impact on the field. A consensus-based taxonomy (Ascertaining Barriers to Compliance; ABC) defined adherence as a temporal sequence of three elements: initiation (taking the first dose), implementation (the extent to which actual use corresponds to prescribed use) and discontinuation (omission of a dose followed by no other doses taken, ending a period of medication persistence) [6]. This taxonomy provides a general framework for adherence research irrespective of the type of data source (e.g. EHD, electronic monitoring, self-report), and therefore requires adaptation to the specific requirements of limitations of the accessible data. Yet, it is largely consistent with terms and definitions proposed by the International Society for Pharmacoeconomics and Outcomes Research (ISPOR) of initial medication adherence [7], adherence (compliance), and persistence [8]. While these definitions represent a common starting point for adherence studies, their application to particular conditions and medications requires further consideration of specific clinical contexts [9]. ISPOR also provides practical guidance on conducting, reporting and evaluating adherence studies using retrospective databases [4]. The ISPOR guideline encourages transparency of adherence measurement and clarity of reporting, and describes common operationalizations and analysis choices. Several methodological studies have detailed and compared alternative operationalizations of adherence elements and offered recommendations for appropriate use [10–17]

Although these recommendations increase standardization and transparency in adherence estimation, they are by necessity formulated at a general level and may be implemented differently in data analysis. Accessible algorithms for computing adherence would represent a much-needed practical support for adherence researchers, but these have been either developed ad-hoc and often not fully disclosed at publication, or developed for proprietary software [18–21]. The field would therefore benefit from open-source algorithms sufficiently comprehensive and flexible to support the whole data analysis process and allow transparent decision-making and data sharing. We developed *AdhereR* in order to provide this support for computing EHD-based adherence (implementation) and persistence estimates within the widely-used open-source environment R [22]. As R has become the preferred statistical software in many research settings, our solution allows researchers to perform complete analyses in R using *AdhereR*, possibly in combination with other R packages, starting from importing raw data, through generating descriptive statistics, interactive exploratory plots, publication-quality figures, and up to modeling of relationships with available predictors or outcomes. Thus, researchers are now able to make informed analysis choices, produce sensitivity analyses, and report the entire process transparently, encouraging an open approach to science and replicability of processes and results.

Moreover, most EHD adherence studies are performed on large samples and do not offer insights into temporal adherence patterns for individual patients, although these are highly informative for data cleaning, hypothesis generation, as well as feedback for clinical decision-making and patient behavior change. Visualizations of individual patterns have been implemented as part of EMR software in some clinical decision support systems (CDSS) [23] although others include only numeric adherence estimates [24], and visualizations are rarely integrated in EHD adherence research [25]. To address this issue, *AdhereR* includes interactive visualizations of individual medication histories and plotting of multiple histories. These facilitate data exploration, decisions regarding clinically-meaningful adherence calculations, and including illustrative examples in analysis reports and publications. Due to the open nature of *AdhereR*, these visualizations may also represent a starting point for developing tools for integrating such visualizations of medication histories in CDSS.

We begin by describing the terms and definitions used by *AdhereR*. We then illustrate the *AdhereR* functions for computing persistence and adherence(implementation) and how they can be used, and describe the visualization tools and their possible applications. Finally, we discuss the potential benefits of *AdhereR* for adherence research, and future areas of improvement and application.

## Terms, definitions and basic considerations

*AdhereR* uses the ISPOR terms of ‘adherence’ (implementation in the ABC taxonomy) and ‘persistence’ to denote the two components of adherence to medications implemented. It is designed to process a single data source and assumes that the medication studied was prescribed to all patients selected for fixed dosing regimens for a period equal to or longer than the time period investigated and that at least one *medication event* was recorded in that period for each patient. *Medication events* are individual records of prescribing or dispensing a specific medication for a patient at a given date. A record needs to include a *patient unique identifier*, an *event date*, and a *duration* (the number of days this quantity would last if used as recommended). *Duration* may be already available or computed based on *quantity* (the number of doses prescribed or dispensed on that occasion) divided by *daily dosage* (the number of doses recommended to be taken daily). Information on *daily dosage* and *medication type* (researcher-defined classification depending on study aims, e.g. based on therapeutic use, mechanism of action, chemical molecule or pharmaceutical formulation) is optional. *AdhereR* is thus designed to be used after data extraction and preparation. For advice on these preliminary steps, we recommend referring to existing guidelines (e.g., [4,5]) and database-specific documentation. We describe the time period investigated using two terms: *follow-up window* (*FUW*; the total period for which relevant medication events are recorded for included patients), and *observation window* (*OW*; the period within the follow-up window for which adherence is computed). For example, a 10-year *FUW* can be extracted from a EHD for patients with long-term treatment; within it, multiple adherence values can be computed for each patient for various *OWs*.

For demonstration and testing, *AdhereR* includes a hypothetical dataset of 1080 *medication events* involving 100 patients over a 2-year *FUW*. Five variables are available: patient unique identifiers (PATIENT_ID), event date (DATE; from 6 July 2030 to 3 September 2044), daily dosage (PERDAY; median 4, range 2–20 doses per day), medication type (CATEGORY; 50.8% medA and 49.2% medB), and duration (DURATION; median 50, range 20–150 days). The timing and characteristics of these events have been formulated to represent various adherence patterns that can illustrate the impact of different analysis choices on results. To facilitate the presentation, we will use two example patients; a subset of the dataset with all medication events related to these two patients is shown in Table 1, and illustrates the file format required as input for AdhereR analyses. These patients have 8 and 11 medication events related to two medication types; patient 37 keeps the same daily dosage but changes event duration with medication change, while patient 76 has three daily dosage changes and two duration changes. We will next illustrate the various options for computing persistence and adherence for these patients (see S1 File for corresponding R script).

**Table 1:** Two example patients (from the hypothetical dataset available in *AdhereR)* used in this article to illustrate computations of persistence and adherence.

PATIENT_ID	DATE	PERDAY	CATEGORY	DURATION
37	04/10/2036	4	medA	50
37	07/30/2036	4	medA	50
37	09/15/2036	4	medA	50
37	01/02/2037	4	medB	30
37	01/31/2037	4	medB	30
37	05/09/2037	4	medB	30
37	08/13/2037	4	medB	30
37	11/09/2037	4	medB	30
76	12/13/2035	20	medA	30
76	01/18/2036	20	medA	30
76	01/23/2036	2	medA	60
76	04/25/2036	2	medA	60
76	08/08/2036	2	medA	60
76	10/03/2036	2	medA	60
76	11/29/2036	2	medA	60
76	12/21/2036	6	medB	30
76	01/05/2037	6	medB	30
76	07/13/2037	6	medB	30
76	10/11/2037	2	medA	30

## Persistence estimates—Treatment episode duration

Persistence is commonly operationalized as a time-to-event variable and analyzed via survival analysis; data summaries include median persistence and proportion of persistent patients at a given moment [6]. Yet, the operationalization of persistence depends on the duration prescribed for achieving the desired therapeutic benefit. For medications prescribed for limited time (acute care, randomized controlled trials), discontinuation occurs frequently as a single event after which no medication is administered until a clinically-relevant or research-related time point (e.g. the duration considered necessary for clinical benefit); in contrast, for long-term treatment (symptomatic treatment in chronic conditions, preventive long-term regimens), the same medication can be discontinued and re-initiated multiple times [9]. Pharmaco-epidemiologic research uses the concept of *treatment episode* to denote a period of active medication use: two consecutive medication events are considered to belong to the same episode if the time difference between the start of the second and the end of the supply from the first does not exceed a researcher-defined *gap length* [12].

Our implementation is encapsulated in the function compute.treatment.episodes(). The user can specify the *FUW start and duration*, and various *gap length* values (in days, weeks, months, years, or percent of *duration*), and choose several computation options: carry over surplus medication from earlier overlapping events within the *FUW*/*OW*; apply carry over only for the same medication type; account for changes in daily dosage in carry over calculations; and consider a change in medication type as new treatment episode. The function outputs a list of all identified treatment episodes for all patients, with start and end dates, duration in days, and gap days corresponding to the last event at the end of the episode (which can be included in the episode if less than the permissible gap length, or otherwise represent a treatment interruption until the next episode or OW end). For longer *FUW* and shorter *gap lengths*, several treatment episodes per patient may result. For shorter *FUW* and more permissible *gap lengths*, most patients will have one episode. The user may opt to further analyze all treatment episodes, or select the first or last treatment episode for time-to-event analyses and descriptive summaries. Results for three parametrizations are presented in Table 2. The first parametrization (90-day gap) produces one episode for patient 37, and two episodes for patient 76. When *gap length* reduces to 60 days, patient 37 has four episodes while patient 76 results remain unchanged. When *gap length* is highly permissive (180) but medication changes are considered new episodes, these patients have two and three episodes, respectively.

**Table 2:** Treatment episodes for two example patients under three different scenarios.

Scenario	Patient ID	Episode number	Date of episode start	Number of gap days after or at the end of the episode	Number of days in the episode (duration)	Date of episode end
A	37	1	2036-04-10	122	608	2037-12-09
	76	1	2035-12-13	144	434	2037-02-19
		2	2037-07-13	32	152	2037-12-12
B	37	1	2036-04-10	61	50	2036-05-30
		2	2036-07-30	67	216	2037-03-03
		3	2037-05-09	66	30	2037-06-08
		4	2037-08-13	122	118	2037-12-09
	76	1	2035-12-13	144	434	2037-02-19
		2	2037-07-13	32	152	2037-12-12
C	37	1	2036-04-10	56	211	2036-11-07
		2	2037-01-02	122	463	2038-04-10
	76	1	2035-12-13	0	374	2036-12-21
		2	2036-12-21	60	234	2037-08-12
		3	2037-10-11	32	62	2037-12-12

A: 90-day gap, no change of treatment episode when medication changes;

B: 60-day gap, no change of treatment episode when medication changes;

C: 180-day gap, new treatment episode when medication changes

All three scenarios refer to a 2-year follow-up window from the first medication event, with carry-over within the observation window, only for the same medication, considering dosage change.

## Adherence (implementation) estimates—Continuous medication availability

The broad operationalization of EHD-based adherence (implementation) is the ratio of medication supplied versus medication prescribed in a time interval, assuming the medication supplied is used. Several methodological articles showed that this apparently simple calculation is applied via numerous algorithms that often produce diverging results, and offered recommendations for appropriate choices [10,11,13–17,26]. Although algorithm descriptions are relatively consistent in these articles, most are rather general. One notable exception is represented by Vollmer and colleagues [11], who described eight variants of continuous multiple-interval measures of medication availability/gaps (CMA) and compared their performance in the context of randomized controlled trials. They are defined by four parameters: 1) how the *OW* is delimited (whether time intervals before the first event and after the last event are considered); 2) whether CMA values are capped at 100%; 3) whether medication oversupply is carried over to the next event interval; and 4) whether medication available before a first event is considered in supply calculations or *OW* definition. These CMA variants can be mapped onto commonly reported metrics used in both experimental and observational studies, such as Medication Possesion Ratio (MPR, corresponding to CMA1 and CMA2) or Proportion of Days Covered (PDC; often used to describe variants from CMA3 to CMA 6). While such common labels are often used without clear descriptions of actual calculations, Vollmer and colleagues provide explicit instructions on computation. We therefore implemented these eight variants, and also added a new alternative computation applicable to repeated adherence estimates in longitudinal cohort studies. These CMA variants can be computed for the whole *OW* (simple-CMA), for each treatment episode within an *OW* (CMA-per-episode), or for repeated sliding windows within the *OW* (sliding-window-CMA). Table 3 presents the nine CMAs, with the values calculated for patient 76. We first provide a short description of each CMA in column 2. All CMAs share the definition of the FUW and OW, while 5–9 use two additional parameters: application of carry over for same type of medication only, and calculation of carry over taking into account dosage changes (both set as FALSE here). Columns 3–6 contain, in sequence, the simple CMA for the example patient 76 in four scenarios: for a 2-year OW, the simple CMA for a 1-year OW, the CMA per episode (in this case two episodes of 479 and 152 days, respectively, one per line) and the sliding window CMA (here two 1-year consecutive windows, one per line).

**Table 3:** The nine CMAs implemented in *AdhereR* (the first eight described in Vollmer et al (11), the last original).

CMA	Description	2-year simple CMA	1-year simple CMA	CMA per episode	Sliding window CMA
1	total number of days of medication supplied in the OW, excluding the last event; the durations of all events are added up, possibly resulting in an estimate > 1.0	67.4%	140.0%	100.3%	85.2%
	$\frac{\#\;days\;supply\;excluding\;last\;event}{first\;to\;last\;event}$			33.3%	30.6%
2	total number of days of medication supplied in the OW, including the last event; the durations of all events are added up, possibly resulting in an estimate > 1.0	65.8%	77.9%	87.7%	98.6%
	$\frac{\#\;days\;supply\;including\;last}{first\;event\;to\;OW\;end}$			39.5%	33.7%
3	CMA1, capped at 1	67.4%	100%	100.0%	85.2%
				33.3%	30.6%
4	CMA2, capped at 1	65.8%	77.9%	87.7%	98.6%
				39.5%	33.7%
5	number of gap days for all event intervals are extracted from the total time interval; (accounting for carry over within OW and excluding the supply left)	67.4%	100%	84.8%	83.2%
	$\frac{\#days\;of\;theoretical\;use}{first\;to\;last\;event}$			33.3%	30.6%
6	number of gap days for all event intervals are extracted from the total time interval; (accounting for carry over within OW and excluding the supply left)	65.8%	77.9%	87.7%	83.8%
	$\frac{\#\;days\;of\;theoretical\;use}{first\;event\;to\;OW\;end}$			39.5%	33.7%
7	number of gap days for all event intervals extracted from the total time interval; (accounting for carry over from before the OW and within OW, and excluding the supply left at the OW end)	65.8%	69.0%	87.7%	83.8%
	$\frac{\#\;days\;of\;theoretical\;use}{OW\;start\;to\;OW\;end}$			39.5%	47.7%
8*	number of gap days for all event intervals extracted from the total time interval; (accounting for carry over within OW and excluding the supply left at the OW end); the period covered by the supply carried-over from before the OW is excluded by a lagged start of the OW	65.8%	68.0%	87.7%	83.8%
	$\frac{\#\;days\;of\;theoretical\;use}{lagged\;OW\;start\;to\;OW\;end}$			39.5%	38.6%
9^#^	Similar to CMA7 and CMA8, except how carryover from before the OW and supply left at the OW end are treated: the supply of each medication event is evenly spread until the next event (ratio days supply up to 100%); oversupply is carried over to the next event	65.8%	70.6%	87.7%	83.8%
	$\frac{\#\;OW\;days,\;each\;weighted\;by\;its\;ratio\;days\;supply}{OW\;start\;to\;OW\;end}$			39.5%	47.7%

CMA: continuous multiple-interval measures of medication availability/gaps; OW: observation window; FUW: follow-up window;

* CMA8 is designed for when an event with a hypothesized causal effect on adherence happens at the OW start (e.g. enrolment in an intervention study); in this case, it may be that the existing supply is not part of the relationship under study (e.g. it delays the actual start of the study for that participant) and needs to be excluded by shortening the time interval examined;

^#^ In longitudinal studies with multiple adherence measures, the assumption of 100% adherence until current supply ends (used in CMA7) may introduce additional variation in adherence estimates depending on where the OW start is located between last event before OW start and the first event in the OW: an OW start closer to the first event in the OW generates lower estimates for the same number of gap days between the two events. To address this, CMA9 first computes a ratio of days’ supply for each event in the FUW (until the next event or FUW end), then weighs all days in the OW by their corresponding ratio to generate an average CMA value for the OW.

### Simple-CMA

Each simple-CMA is encapsulated in a function CMA*n*(), where *n* is a number between 1 and 9, returning an object that can intelligently print itself (as plain text, Markdown or LaTeX), plot itself, and that contains the CMA estimates per patient, the primary event data, and the parameter values chosen. For all functions, the user can specify the *FUW* and *OW start* and *duration* (*OW* can be equal to *FUW*, or a time interval within the *FUW*). Some calculations carry over surplus medication from earlier overlapping events within the *OW* (for CMA5 and higher), and from before the *OW start* (for CMA7 and higher; three different adjustments, described in Table 3). Two extra options can apply from CMA5 upwards to both carry-over adjustments: apply carry-over only for the same medication type, and consider changes in daily dosage for carry-over calculations. Table 3 illustrates simple CMA values for patient **76** in two scenarios: a 2-year *OW* (equal to *FUW*), and 1-year *OW* (months 6–18 in a 2-year *FUW*). CMA values are similar for a 2-year OW, as only the OW definition changes the calculation (i.e. until last event versus OW end). By contrast, the 1-year OW generates CMAs ranging from 68% to 140%, as several parameters influence the calculation. These differences highlight the importance of choosing parameter values based on their suitability for the clinical context and study design, clearly justifying choices, performing sensitivity analyses, and reporting the whole process transparently.

### CMA per episode

Non-persistence and adherence have been usually reported as alternative calculations for the same time interval (e.g. [27]). The ABC and ISPOR taxonomies show they are conceptually different, and advise calculating adherence (implementation) only in periods of persistent use. Studies that apply this conceptual distinction show that implementation may be substantially higher during persistent medication use [28–31]. Research on this topic is incipient, and more evidence is needed on the differential contributions of adherence and persistence in specific clinical contexts. To facilitate transparent comparisons of these approaches, we developed the function CMA_per_episode(), which allows the computation of all simple-CMAs (with applicable options) per treatment episode (defined as described above). In contrast to the simple-CMAs, this function returns the list of treatment episodes per patient, each with its estimated CMA value. Table 3 includes CMAs 1 to 9 for patient 76 for two treatment episodes (90-day permissible gap). CMAs are consistently higher for the first episode. As all episodes start with an event and end when supply of the last event finishes, only the OW definition, carry-over within OW, and capping at 100% influence the calculation in this case.

### Sliding window CMA

The limitation of CMAs-per-episode is that they assume a sudden shift from persistence to discontinuation once the supply from the last event in an episode is finished after being used as prescribed and the time interval until the next event is longer than the permissible gap. However, patients may use less medication over longer periods of time at the end of an episode, and, from this perspective, this end is unlikely to be an abrupt interruption but rather a slow decrease in the likelihood of actively using medication. We offer an alternative operationalization that relaxes this assumption in the CMA_sliding_window() function. It computes any of the simple-CMAs in sliding windows of a user-defined fixed size (successive or overlapping windows). The beginning, duration and step of the sliding windows can be specified (as fixed durations or as total number of sliding windows within the OW). Like CMA_per_episode(), this function returns all sliding windows per patient with their corresponding CMAs. If the OW duration equal to the permissible gap in a CMA-per-episode, OWs completely outside treatment episodes would have 0% CMA and represent treatment interruptions (non-persistence). If however they overlap with the end of an episode, successive OWs would indicate decreasing CMAs. Table 3 shows CMA 1 to 9 for patient 76 for two sliding windows (1-year duration, 1-year step duration). Similar to CMA_per_episode, the first window has consistently higher values than the second. The differences with CMA-per-episode are explained by the different time intervals covered (1-year sliding-windows, versus 479- and 152-day episodes), and by the relevance of carry-over from outside the OW in sliding-window-CMA.

## Visualization of medication histories and adherence estimates

Meaningful visual exploration is essential for both data analysis and results dissemination, particularly when complex decisions are needed, as with understanding adherence to medications. *AdhereR* implements high-quality, intuitive and informative plotting that highlights the essential parameters and entities involved in estimating adherence. All CMAs (1 to 9; simple, per-episode and sliding-window) can intelligently plot themselves either as interactive support for exploratory analyses or as publication-quality figures.

### Interactive visualization of individual medication histories

**Fig 1:**
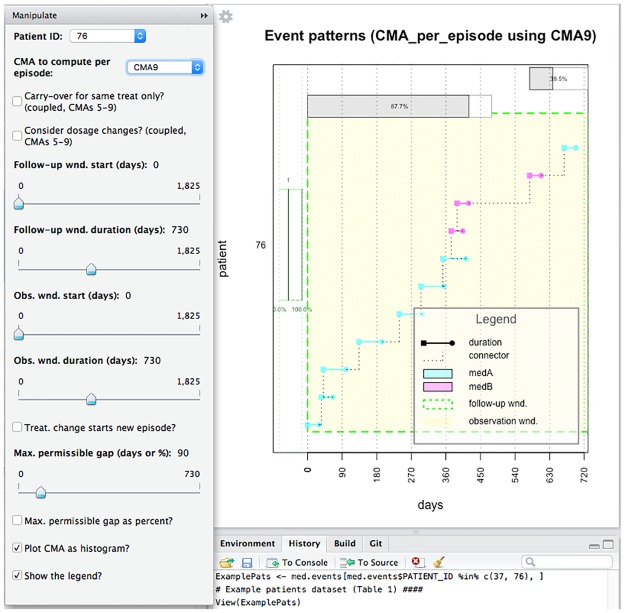
Screenshot of interactive plotting session for patient 76.

For exploratory purposes we have implemented interactive plotting of medication histories and CMA estimates (simple, per-episode, or sliding-window) within RStudio (https://www.rstudio.com/). The user can select a patient (drop-down list of unique identifiers), a CMA (1 to 9), and change various parameters applicable to the *FUW* and *OW*, CMA, treatment episode, or sliding window. The effects of these choices on the CMA computation and values are visualized in real time (Fig 1). Depending on the complexity of the computation and the hardware, this might be more or less instantaneous (see software performance benchmarks in the following section).

### Publication-quality plotting of medication histories

**Fig 2:**
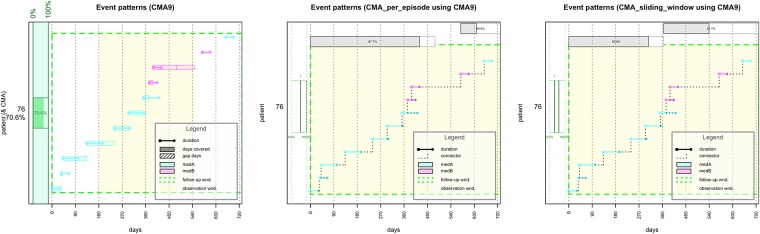
Publication-quality plotting for simple, per-episode, and sliding-window CMA 9 for patient 76.

Individual medication histories and CMA calculations can also be plotted at publication quality as PDF, (E)PS, TIFF, SVG, or other R-supported formats), controlling for various graphical parameters such as colors and line style for various components (grayscale if needed); drawing and attributes of CMA, FUW, OW, and legend; and display of time (days since a relevant date or calendar dates). For CMAs-per-episode and sliding-window-CMA, multiple episodes or windows can be displayed for a given patient stacked above the medication history plot as individual bars. The start and end of the episode/window are plotted with corresponding CMA values, and the CMAs distribution per patient is displayed on the side. A plot can also include several patients, allowing meaningful comparisons between medication histories and CMA estimates. A special parameter allows the medication histories to be aligned on the horizontal axis at the first event per patient, facilitating comparability. Fig 2 shows CMA 9 (simple, per-episode and sliding-window) for patient 76.

## Performance on large datasets and software and hardware requirements

*AdhereR* was designed to work with very large datasets on available consumer-level hardware, and it is heavily optimized (the implementation is in pure R) without affecting the clarity of the code. If run on a multi-core/multi-processor machine or computer cluster, it gives the user the possibility to use (completely transparently) two parallel backends: multicore (preferred on Linux, BSD and MacOS but currently not available on Microsoft Windows) and snow (Simple Network of Workstations; available on all platforms), reducing dramatically the computation time. Another goal in designing *AdhereR* was to future-proof it as well as possible and to reduce the number of dependencies to a minimum. Finally, *AdhereR* aims to promote replicable and transparent science and is released under GPL v3 (https://www.gnu.org/licenses/gpl-3.0.en.html) encouraging use and extension in the spirit of open source. For more information on the implementation and performance, please consult the online package help, the vignette and the source code, freely available on CRAN (The Comprehensive R Archive Network; https://cran.r-project.org/) and in the package’s GitHub repository (https://github.com/ddediu/AdhereR).

For example, Table 4 below gives the running times (single-threaded and two parallel multicore threads) for a database of 13922 unique patients and 112984 prescriptions of all CMAs described here on an Apple MacBook Air 11” (7, 1; early 2015) with 8Go RAM (DDR3 @ 1600MHz) and a Core i7-5650U (2 cores, 4 threads with hyperthreading @ 2.20GHz, Turbo Boost to 3.10GHz) CPU, using MacOS X El Capitan (10.11.6), R 3.3.1 (64 bits) and RStudio 1.0.44. Table 5 gives the same information for a very large database of 500,000 unique patients and 4,058,110 prescriptions (generated by repeatedly concatenating the database described above and uniquely renaming the participants) on a mid/high-range desktop computer with 16Go RAM and a Core i7-3770 CPU (4 cores, 8 threads with hyperthreading @ 3.40GHz, Turbo Boost to 3.90GHz), using OpenSuse 13.2 (Linux kernel 3.16.7) and R 3.3.2 (64 bits).

**Table 4:** Performance (single and two-threaded) computing CMAs for a large dataset (13922 patients with 112983 events) on a consumer-grade laptop.

CMA	Single-threaded (min)	Two threads, multicore (min)	Two threads, snow (min)
CMA 1	0.68	0.35	0.37
CMA 2	0.69	0.36	0.41
CMA 3	0.66	0.34	0.38
CMA 4	0.67	0.36	0.38
CMA 5	0.94	0.50	0.53
CMA 6	0.97	0.52	0.54
CMA 7	0.93	0.48	0.51
CMA 8	2.20	1.21	1.19
CMA 9	2.66	1.42	1.44
per episode*	4.40	2.32	2.33
sliding window^#^	10.73	5.80	5.66

* gap = 180 days → 20009 episodes;

^#^ length = 180 days, step = 90 days → 97454 windows

The times shown are “real” (i.e., clock) running time in minutes as reported by R’s system.time() function. In all cases, the follow-up window and observation window are identical and 2 years long. CMA per episode and sliding window computed CMA1 for each episode/window. Please note that the parallel times are longer than half the single-core times due to various overheads.

**Table 5:** Performance (single and four-threaded) computing CMAs for a very large dataset (500000 patients with 4058110 events) on a mid/high range desktop computer.

CMA	Single-threaded (min)	Four threads, multicore (min)	Four threads, snow (min)
CMA 1	30.66	9.62	12.59
CMA 2	29.65	8.17	15.26
CMA 3	28.01	7.64	10.14
CMA 4	29.65	8.15	10.74
CMA 5	41.68	11.39	14.44
CMA 6	43.33	11.91	18.73
CMA 7	41.35	11.32	16.47
CMA 8	99.97 (= 1.7 hours)	25.97	33.66
CMA 9	117.33 (= 1.9 hours)	31.58	50.05
per episode*	192.47 (= 3.2 hours)	50.51	66.57
sliding window^#^	460.86 (= 7.7 hours)	119.97 (= 2.0 hours)	204.81 (= 3.4 hours)

* gap = 180 days;

^#^ length = 180 days, step = 90 days

## Discussion and conclusions

*AdhereR* is, to our knowledge, the first open-source implementation that allows a flexible and comprehensive investigation of EHD-based adherence to medications on consumer-grade hardware and software. It estimates persistence by identifying treatment episodes for each patient and calculating the start date, end date, and duration of each episode. Adherence (implementation) is estimated under three conditions: per observation window (assuming persistence), within each treatment episode (accounting for persistence), and for consecutive sliding windows (with or without overlaps). Interactive and publication-ready plotting is included for visualization of medication events, as well as persistence and adherence estimates. These allow in-depth exploration of longitudinal medication use patterns and visual comparison of the impact of different methods of calculation on persistence and adherence values. *AdhereR* aims to help researchers better understand the data, select clinically-meaningful parameters, document this process, and communicate the results in an open, transparent, and reproducible manner.

Probably the main benefit of *AdhereR* is that it facilitates the sharing of computation methods: authors can upload supplementary materials with analysis codes explaining their choices of parameter values. This much needed increase in methods standardization and transparency is further facilitated in R by recent advances in reproductive data analysis and reporting using *Sweave* [32] or *RMarkdown* [33]. We acknowledge that different medications and health conditions may require different methods and parameter choices; moreover, in many clinical areas it is yet unknown which assumptions are most appropriate. Therefore, our aim is not to prescribe a single ‘best’ method. On the contrary, the highly-parameterized and flexible functions implemented in *AdhereR* are perfectly suited for comparison studies: different parameter values can be selected to test their impact on adherence and persistence values and their relationship with relevant clinical characteristics and outcomes. The visualization functions may also be used for data exploration and hypothesis generation, or for communication purposes (for example in teaching, explaining the calculation methods to collaborators, or disseminating study findings). They can also be integrated in open-source EHD-based clinical decision support systems (CDSS) as part of adherence support interventions.

EHD represents only one of the possible data sources used to assess adherence to medication. Its main advantage is the relatively low cost of obtaining information over large samples of patients across long periods of time, if the routine care of these patients is recorded systematically for medical or financial purposes. By comparison, alternative methods such as electronic monitoring, self-report, or metabolites monitoring are resource intensive to various extents, and require the active involvement of patients in the process of data collection, which may lead to lower response rates and thus reduce generalizability or be altogether impractical in some settings. Moreover, relying on routine data collection limits measurement biases related to study participation. On the other hand, EHD can only be used retrospectively and has lower temporal granularity, as it relies only on prescribing and/or dispensing information and on the fundamental assumption that medication supplied is used. Therefore, the choice of EHD as the preferred data source for measuring adherence in a specific context needs to be based on careful consideration, and triangulated with alternative methods where possible [1, 3].

The current version of *AdhereR* has several limitations that could eventually be addressed in future versions and/or extensions. First, it is currently designed for a single data source, and thus does not compute (time to) medication initiation, or adjustment for prescription patterns [34] or hospitalizations [16]. Such computations and adjustments can be performed at present by correcting the AdhereR input or output with the available information from the additional data source (e.g. adding the duration of hospitalization to the duration of the ongoing medication event if dates of hospitalization are known). Second, combined adherence estimates for multiple medications proposed in the literature (e.g. [35]) are not implemented; separate estimates for each medication may be more informative if the aim is to explore longitudinal patterns and possible interaction effects on outcomes. If however the intention is to examine the clinical effects of simultaneous use of all medications, the output of existing functions can be used to calculate combined adherence and persistence scores (e.g. computing treatment episodes with 0 permissible gap length may be used to identify days with at least one medication unavailable and calculate a daily polypharmacy possession ratio). Third, we did not yet implement a way of distinguishing therapy switches and concomitant medication use. The visualization tools allow identification of such patterns, and the relevant records can either be excluded from further analyses or adjusted based on available parameters (e.g. carry-over only for the same medication, new treatment episode if medication changes). The current version is sufficiently flexible to offer concrete solutions for various clinical scenarios, and we would welcome feedback and questions on its application or development for less common scenarios.

Developing a strong science on adherence to medications requires sustained improvement of methodological rigor, transparency, and replicability. Open-source software has been a catalyzer of this progress in many scientific fields. We hope that *AdhereR* will support this process adherence research and invite the research community to contribute to its improvement and further development.

## Supporting information

S1 File AdhereR demonstration R script. (R)
